# An ultrasensitive detection platform for cocaine: Aptasensing strategy in capillary tube

**DOI:** 10.3389/fchem.2022.996358

**Published:** 2022-10-19

**Authors:** Javad Zamanian, Zahra Khoshbin, Hossein Hosseinzadeh, Noor Mohammd Danesh, Ali Khakshour Abdolabadi, Khalil Abnous, Seyed Mohammad Taghdisi

**Affiliations:** ^1^ Pharmaceutical Research Center, Pharmaceutical Technology Institute, Mashhad University of Medical Sciences, Mashhad, Iran; ^2^ Department of Medicinal Chemistry, School of Pharmacy, Mashhad University of Medical Sciences, Mashhad, Iran; ^3^ Targeted Drug Delivery Research Center, Pharmaceutical Technology Institute, Mashhad University of Medical Sciences, Mashhad, Iran; ^4^ Department of Pharmacodynamics and Toxicology, School of Pharmacy, Mashhad University of Medical Sciences, Mashhad, Iran; ^5^ Institute of Science and New Technologies, Tehran, Iran; ^6^ Department of Pharmaceutical Biotechnology, School of Pharmacy, Mashhad University of Medical Sciences, Mashhad, Iran

**Keywords:** aptamer, cocaine, aptasensor, fluorescence microscopy, capillary tube, on-site detection

## Abstract

Cocaine as a detrimental addictive drug threats human health through inducing heart problem, blood pressure, anxiety, immunodeficiency, paranoia, and organ damage. Thus, the quantification of cocaine in the biological samples by a simple, high specificity, and fast method is highly urgent to decrease the harmful effect of the misuse of this drug. In this study, we constructed a novel fluorescent aptasensor by combining the fluorescein (FAM)-modified specific aptamer and AuNPs in a capillary tube as the sensing substrate for the first time. The presence of cocaine recovered the fluorescence response of the aptasensor through interaction with the aptamer and differentiation of the aptamer@AuNPs complex. By fluorescence microscopy imaging of the aptasensor substrate and its quantitative analysis, a remarkable linear range from 100 pM to 600 µM and the ultra-low limit of detection (LOD) as 0.31 pM were achieved for the target detection. Cocaine was successfully quantified in the real samples (human serum and urine) by using the aptasensor. The aptasensor is simple, easy-to-use, favorable applicability, and cost-effective; and to the best of our knowledge, it is the first use of the capillary tube as a sensing platform just by using about 3 μl of the samples. It is also an easy-to-carry tool, promising for the on-site target detection. Besides, it can be a portable device for monitoring cocaine by using a handheld single-beam fluorescence microscope. It can be an appropriate detection tool in forensic science and medicine.

## 1 Introduction

Cocaine (benzoylmethylecgonine) is one of the noticeable abuse substances and addictive drugs, illicit in most societies, such as the United States and Europe (Shahdost-Fard and Roushani, 2016). The destructive effects of cocaine emerge in synaptic cleft by blocking the reuptake of some catecholamine, including dopamine and noradrenaline, provokes the sympathetic system, raises heart rate, and finally, induces some disorders, like initial vasodilation and coronary vasoconstriction. Also, the undesirable cardiovascular changes can outcrop in cocaine consumers (Pantaleão et al., 2021). Long-term use of cocaine damages the health care system and stimulates the central nervous system. Misuse of cocaine directly induces some disorders on human body, including anxiety, blood pressure, immunodeficiency, paranoia, organ damage, and so on (Shahdost-Fard and Roushani, 2016; Hashemi et al., 2017). So, it would be necessary to develop new methods for determining cocaine, applicable in forensic science, toxicology, and pharmacology.

After consuming the cocaine, it will rapidly convert to the two major metabolites ecgonine methyl ester (EME) and benzoylecgonine (BE) (Kolbrich et al., 2006) which is more important for Law enforcement authorities to determine the amount of cocaine in order to take rapid and proper decision about the criminal (Eliaerts et al., 2021). Thus, to many methods have been utilized to determine amount of cocaine, such as radioimmunoassay, enzyme-linked immunosorbent assay (ELISA), high performance liquid chromatography (HPLC), and gas chromatography-mass spectrometry (GC-MS) (Abnous et al., 2018). In spite of their accuracy and reliability, some disadvantages like being time-consuming, requiring complicated and expensive instruments, and dependency on skilled operators have reduced the use of these methods (Sanli et al., 2020). The other method like colour tests apart from some advantages such as cost effective, simplicity, detect are limited by some drawbacks like short expiration dates and lack of proper interpretation due to discolouration during time (Eliaerts et al., 2021). Hence, aptamer-based biosensors (aptasensors) have been introduced as the sensitive methods for the cocaine determination with superior diagnostic performance (Neves et al., 2015; Emrani et al., 2016; Wang et al., 2016; Su et al., 2017; Oueslati et al., 2018). Aptamers are the fragment of DNA or RNA oligonucleotides, selected *in vitro* by the SELEX (systematic evolution of ligands by exponential enrichment) process. Their spatial three-dimensional structure enables aptamers to selectively bind to their targets through the hydrogen bonding, electrostatic interaction, π-π stacking, and van der Waals forces (Bozokalfa et al., 2016; Roushani and Shahdost-fard, 2016; Aydindogan et al., 2019; Khoshbin et al., 2021a). Aptamers have superior properties of facile generation, little size, facile modification, cost-effective generation, great binding affinity to targets, non-immunogenicity, reusability, and high stability, making them prominent for developing biosensors compared to antibodies as biorecognition elements (Roushani and Shahdost-Fard, 2015; Taghdisi et al., 2020; Wu et al., 2020).

To now, the different types of nanostructures, such as gold nanoparticles (AuNPs), carbon nanotubes (CNTs), graphene oxide (GO), silicon nanowires, silica nanoparticles (SNPs), and carbon nitride nanosheets, have been assembled by aptasensors to improve their efficiencies, based on their unique advantages of high chemical and thermal stability, regular structure, large surface area, etc. Extensively, AuNPs have been applied to modify aptasensors relying on their superiority, including high quantum yield, large surface-to-volume ratio, great photo-stability, low toxicity, and so on (Khoshbin et al., 2018). Besides, AuNPs have been extremely used as a quencher segment in aptasensors, because of high stability and super-quenching efficiency to suppress fluorescence emission of a wide range of fluorophores (Lin et al., 2011; Wang et al., 2015; Sun et al., 2021).

Today, developing portable aptasensors has attracted a remarkable attention for on-site target detection. Hence, embedding aptasensors on easy-to-carry platforms (e.g., paper, glass slide, plastic sheet, etc.) is of great importance (Khoshbin et al., 2021b). In the present study, a simple portable fluorescent aptasensing platform is designed to monitor ultra-low levels of cocaine by using the internal surface of capillary tube as the aptasensor platform for the first time. In spite of high sensitivity and portability, the developed aptasensor is a cost-effective and rapid detection assay.

## 2 Experimental section

### 2.1 Reagents and materials

(3-aminopropyl) triethoxysilane (APTES), sulfuric acid (98%), hydrogen peroxide (98%), activated carbon (196 ng L^−1^), graphene oxide (GO, 196 ng L^−1^), carbon nanotube (CNT, 196 ng L^−1^), AgNPs (0.05 g L^−1^), CuNPs (0.58 g L^−1^), SNPs (196 ng L^−1^), and capillary tube were supplied from Sigma-Aldrich (United States). Glutaraldehyde was purchased from Merck Company. Addictive drugs, including morphine, tramadol, methadone, ibuprofen, diazepam, and lorazepam were procured from Sigma-Aldrich (United States).

The DNA aptamer strand was purchased from Microsynth Company (Switzerland) with the sequence of 5′-NH_2_-GGC-GAC-AAG-GAA-AAT-CCT-TCA-ACG-AAG-TGG-GTC-GCC-FAM-3′ (Stojanovic et al., 2001; Neves et al., 2010a; Bottari et al., 2020). The aptamer possesses the binding affinity of (0.3 ± 0.1) μM to the target (Neves et al., 2010b). The aptamer sequence was diluted with Tris-HCl buffer (20 nM, pH = 7.5). Solution preparation was done by deionized water in all processes. The capillary tube was chosen with following features 75 mm length, I.D. 1.1–1.2 mm, O.D 1.5–1.6 mm. Human serum and urine were supplied from healthy volunteer.

### 2.2 Apparatus

The fluorescence microscopic images of the capillary tubes were observed by using an inverted Olympus fluorescence microscope IX53, equipped with DP73 microscope digital camera Zoom: 4x, Angle: 90°. The excitation and emission wavelengths of the FAM molecules are 495 and 517 nm, respectively.

All the images were analyzed by ImageJ software to determine the mean brightness values and surface plots.

The size, zeta potential, and morphology of AuNPs were analyzed by a particle size analyzer (Malvern, United Kingdom) and transmission electron microscopy (TEM), respectively (CM120, Philips, Holland). HPLC was done by Knauer chromatography system equipped with Knauer Solvent Organizer K-1500, WellChrom HPLC Pump K-1001, and WellChrom UV Detector K-2600.

### 2.3 Gold nanoparticles preparation

AuNPs were synthesized by the chemical reduction of HAuCl_4_ in the presence of sodium citrate, according to the classical method (Storhoff et al., 1998). The citrate-stabilized AuNPs were centrifuged at 10,000 g for 20 min at 4°C, followed by elimination of supernatant. Then, the synthesized AuNPs were resuspended in ultrapure water. Extinction coefficient of 2.7 × 10^8^ M^−1^ cm^−1^ at *λ* = 520 nm was used to determine the concentrations of AuNPs.

### 2.4 Cleaning internal surface of capillary tube

First, the capillary tubes were immersed in methanol (98%) to remove the probable pollutions. Then, all the cleaned tubes were immersed in the piranha solution, including hydrogen peroxide and sulfuric acid with a proportion of 1:4 for 1.5 h at 60°C. All the tubes were washed by ethanol and deionized water, dried under the nitrogen flow, and heated in an oven at 60°C for 2 h that activated the internal surface of the capillary tubes by forming the hydroxyl groups (Wijesinghe et al., 2020).

### 2.5 Modifying internal surface of capillary tube

The activated internal surface of the capillary tube was silanized by injection of APTES solution with the concentration of 1.8% (v/v) and incubation at room temperature for 30 min. The solution was drained, and the tube remained at room temperature for 4 h. Then, it was washed with ethanol and deionized water several times. Finally, the tube was dried by using a slow flow of nitrogen gas and annealed in a high-temperature oven (110°C) for 2 h (Wijesinghe et al., 2020).

The glutaraldehyde solution with the concentration of 1.8% (v/v) was injected into the APTES-modified tube and incubated for 1 h. Then, it was washed by deionized water and dried under a slow nitrogen flow.

### 2.6 Aptamer immobilization

3 μl of the diluted aptamer solution by Tris-HCl buffer (500 nM) (pH = 7.4) was injected into each tube and incubated at room temperature for 2 h to bind to the immobilized aldehyde group on the internal surface of the capillary tube through the covalent binding. Finally, the aptamer solution was drained; and the capillary was washed by deionized water and then, dried by a slow flow of nitrogen.

### 2.7 Gold nanoparticles incubation

3 μl of AuNPs were injected into all tubes and incubated for 105 min at room temperature to quench the fluorescence emission of the FAM molecule. Then, the NPs were drained; and the tubes were rinsed by deionized water, and subsequently dried under a slow nitrogen stream, obtaining the ready-to-use aptasensor on the internal surface of the capillary tube.

### 2.8 Cocaine quantification

To determine the amount of cocaine, the different concentrations of cocaine (diluted by deionized water) were injected into the modified capillary tubes and incubated for 1.5 h at room temperature. After that, the target solution was drained; and the capillary tube was rinsed by deionized water and dried under a slow nitrogen stream. The microscopy images of the tubes were recorded by Olympus fluorescence microscope, followed by the quantitative analysis by using ImageJ software.

### 2.9 Aptasensor specificity

To assess the selectivity of the aptasensor, the different types of the addictive drugs, including morphine, tramadol, methadone, ibuprofen, diazepam, and lorazepam were tested against the target (the final concentration of the all samples were 8 µM).

### 2.10 Real sample analysis

To determine the practical efficiency of the aptasensor, the determination of the target was tested in the real samples, like human serum and urine. For this purpose, the serum and urine samples were diluted by PBS and Tris-HCl buffer 100 and 50 times, respectively. Next, we prepared a stock solution of cocaine by dissolving it in the diluted buffer. Then, the different concentrations of cocaine solution were prepared by using the stock solution. The recovery values of the real samples were calculated by dividing the found concentration of cocaine to the spiked concentration.

## 3 Results and discussions

### 3.1 Sensing mechanism of aptasensor

**FIGURE 1:**
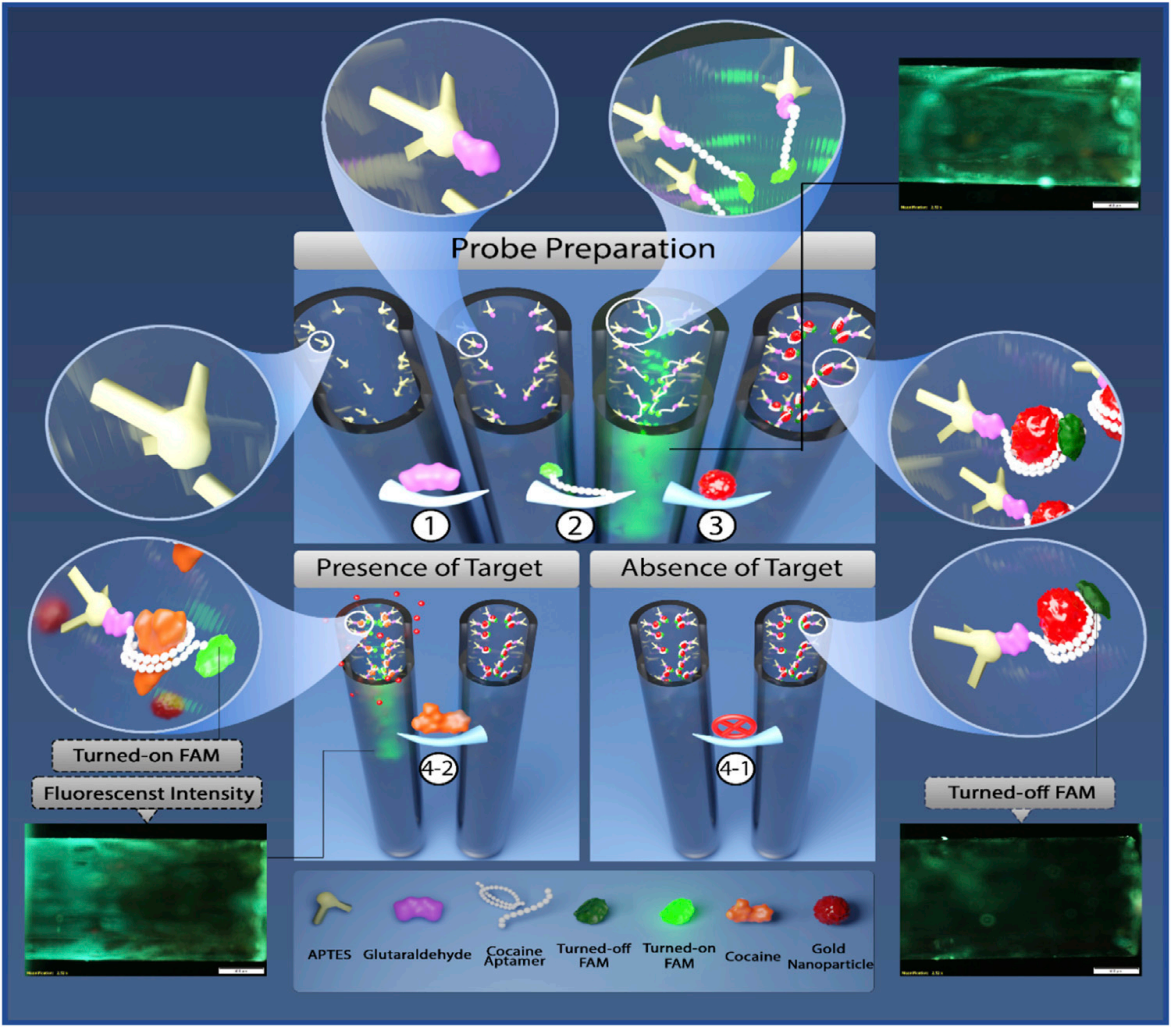
The schematic illustration of the designed aptasensor for the cocaine detection.

Figure 1 indicates the mechanism of the designed aptasensor. Based on Figure 1, the FAM-labeled aptamer strands could bind to the internal surface of the silanized capillary tube through interaction between the amine group at its 5′-end and glutaraldehyde cross-linker. Then, AuNPs were injected into the tube that quenched the fluorescence emission of the aptamers through the surface electron transfer (SET) mechanism (Chen et al., 2010). By adding cocaine, AuNPs were substituted with cocaine due to the high binding tendency of the aptamer to the target that regained the fluorescence intensity of the FAM-labeled aptamer. The formation of the aptasensor was proved by gel electrophoresis (2.5% agarose). Supplementary Figure S1 displays the shining band related to the aptamer, while no band emerged for AuNPs and aptamer@AuNPs complex (lanes 1, 2, and 3, respectively). It confirmed the aptamer interaction with AuNPs and prosperous construction of the aptasensor.

### 3.2 Optimization of effectual parameters on aptasensing platform

#### 3.2.1 Optimization of glutaraldehyde/(3-aminopropyl) triethoxysilane volumetric ratio

To achieve the highest efficiency of the aptasensor, it is required to the binding sites for the attachment of the highest number of the aptamer strand on the internal surface of the capillary tube. Hence, the different concentrations of APTES [1.8, 3.6, and 5.4% (v/v) in ethanol, 3 μl] and glutaraldehyde [0.9, 1.8, and 2.7% (v/v) in deionized water, 3 μl] were tested to gain their optimum values for attaining the maximum performance of the aptasensor. The fluorescence microscopic images of the capillary tubes containing the specific aptamer (500 nM, 3 μl) and the different concentrations of APTES/glutaraldehyde were observed by Olympus fluorescence microscope, recorded by digital camera, and quantitatively analyzed by ImageJ software. Supplementary Figure S2A illustrates the fluorescence images of the tubes, and their quantitative analysis (mean brightness values) and corresponding surface plots, done by ImageJ software. Based on Supplementary Figure S2A, the highest fluorescence response of the aptasensor was obtained for 1.8% (v/v) of APTES and 1.8% (v/v) of glutaraldehyde. The assays were repeated for three times; and the results are presented in Supplementary Figure S2B. The obtained results proved 1.8% (v/v) of APTES and 1.8% (v/v) of glutaraldehyde as the optimum values, considering for all subsequent assays.

#### 3.2.2 Optimization of specific aptamer concentration and incubation time

The various concentrations of the aptamer (100, 200, 500, and 700 nM) were applied to construct the aptasensor; and the optimum concentration was determined as one that provided the highest fluorescence emission. Supplementary Figure S3A indicates the fluorescence images of the tubes, corresponding brightness parameter, and surface graphs. Based on Supplementary Figure S3A, the brightness of the capillary tube increased by enhancing the aptamer concentration to 500 nM. Since the brightness value possessed no considerable increase for the aptasensing platform containing 700 nM of the aptamer compared to that for 500 nM, the optimum concentration of the aptamer was determined 500 nM. The assays were repeated for three times that proved the result (Supplementary Figure S3B). The optimum incubation time of the aptamer was also determined as depicted in Supplementary Figure S4A. Supplementary Figure S4B represents the plot of the mean brightness value against the incubation time of the aptamer with three repetition of the experiments, highlighting 2 h as the optimum incubation time.

#### 3.2.3 Optimization of fluorescence quencher

To achieve the highest fluorescence quenching of the FAM-labeled aptamer, the diverse quenchers, including activated carbon, SNPs, CuNPs, GO, AgNPs, CNT, and AuNPs were examined. All NPs were provided from Sigma-Aldrich except AuNPs (synthesized by the chemical reduction of HAuCl_4_) (Storhoff et al., 1998). The particle size and zeta potential for the citrate-coated AuNPs were 14.2 ± 1.2 nm and −31.4 ± 2.8 mV, respectively. Supplementary Figure S5 indicates the TEM analysis of AuNPs that highlights their well-dispersed state with a diameter about 12 nm. Supplementary Figure S6A displays the fluorescence images of the modified capillary tubes in the presence of the different NPs. Based on Supplementary Figure S6A, the fluorescence emission of the aptamer possessed the highest reduction in the presence of AuNPs. Hence, AuNPs were chosen as the most appropriate quencher for doing all assays. Supplementary Figure S6B quantitatively indicates the obtained results with three repetitions.

To determine the optimum concentration of AuNPs, the different concentrations (0.2, 0.4, 0.6, and 1 nM) were injected into the aptamer-modified tubes. The recorded fluorescence images and related quantitative data are illustrated in Supplementary Figures S7A,B, respectively. The results proved 1 nM as the optimum concentration of AuNPs that achieved the highest fluorescence quenching of the aptamer. Besides, the incubation time of AuNPs was optimized as illustrated in Supplementary Figures S8A,B. The results confirmed 105 min as the optimum incubation time of AuNPs.

#### 3.2.4 Optimization of cocaine incubation time

To obtain the optimum incubation time of the target, cocaine (600 μM, 3 µL) was injected to the aptasensor and incubated at the different times. Supplementary Figure S9A displays the fluorescence images of the aptasensor, corresponding brightness values, and surface plots. The quantitative data for the three repeats of the assays are collected in Supplementary Figure S9B. The results highlighted 1.5 h as the optimum time of the cocaine incubation.

### 3.3 Target detection by aptasensor

**FIGURE 2:**
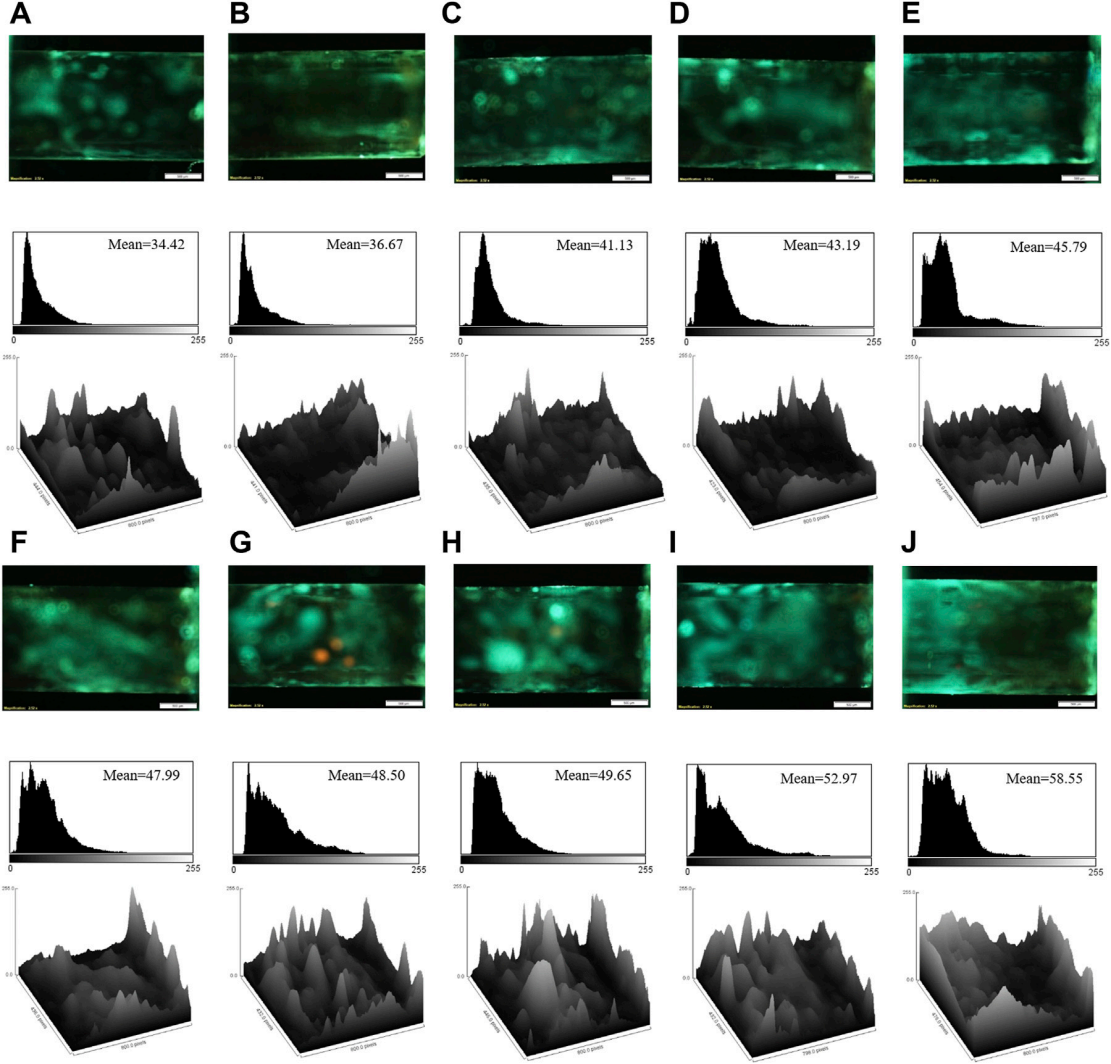
The fluorescence images, corresponding mean brightness values, and surface plots of the aptasensor in the presence of the different concentrations of cocaine: **(A)** 100 pM; **(B)** 500 pM; **(C)** 10 nM; **(D)** 50 nM; **(E)** 250 nM; **(F)** 2 μM; **(G)** 4 μM; **(H)** 8 μM; **(I)** 50 μM; **(J)** 600 μM. Scale bar: 500 μm.

**FIGURE 3:**
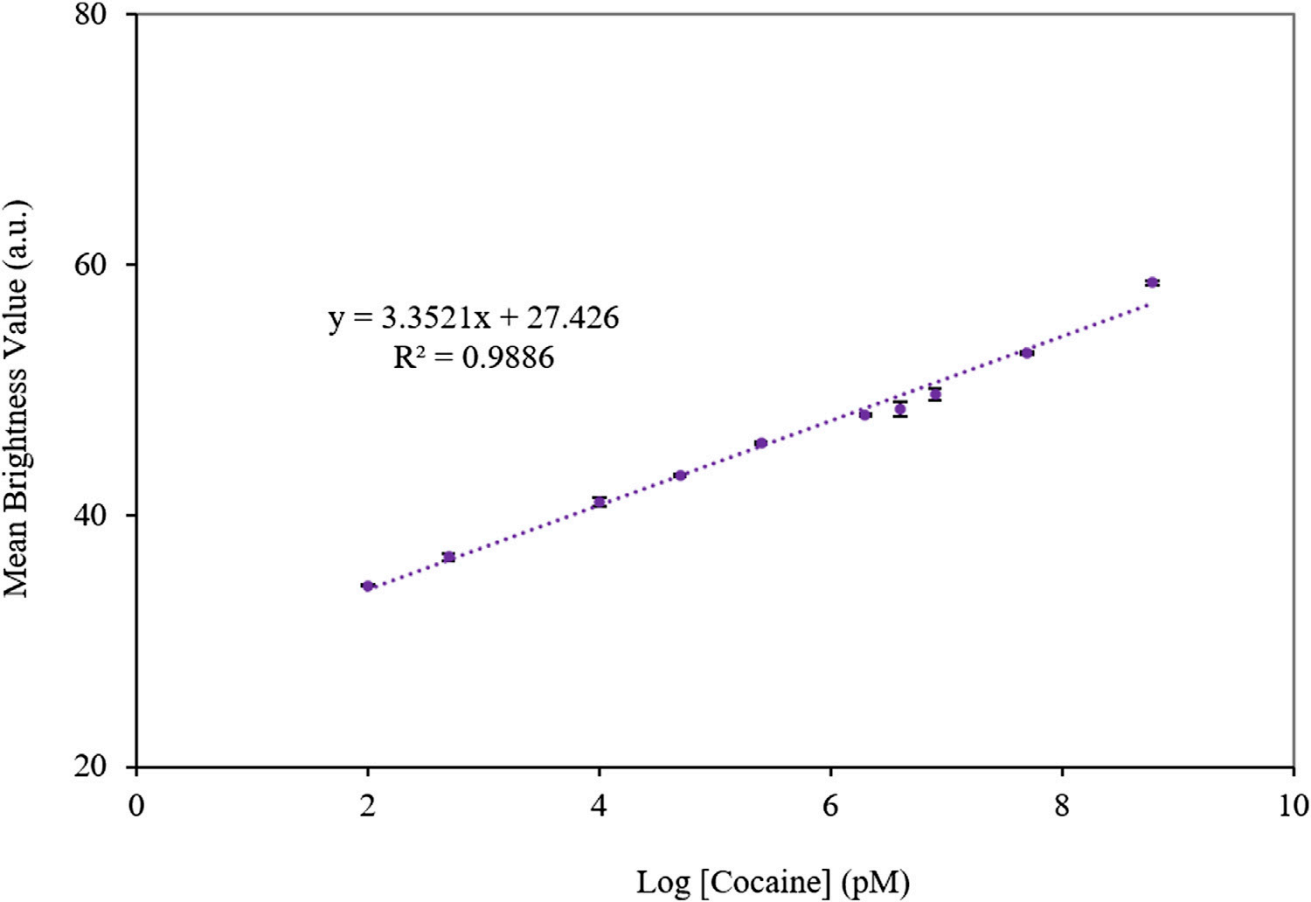
The calibration curve of the mean brightness value and cocaine concentration based on the fluorescence response of the aptasensor. The error bars represent the average standard errors for three measurements.

To detect the target by the developed aptasensor, the various concentrations of cocaine were injected into the modified capillary tubes. Figure 2 illustrates the microscopic images of the capillary tubes and related quantitative analysis. Based on Figure 2, an increase in the cocaine concentration enhanced the fluorescence emission of the aptasensor, due to the high affinity of the aptamer to cocaine and replacement of AuNPs by cocaine in the aptamer@AuNPs complex. Considering mean brightness value as a criterion for the fluorescence emission of the aptamer, it possessed a linear relationship with a wide range of the cocaine between 100 pM and 600 µM (Figure 3). Besides, the detection limit (LOD) was computed as 0.31 pM based on the fluorescence response of the aptasensor by calculating 3 × Slope^−1^ × Blank Standard deviation (Schmitteckert and Schlicht, 1999). Remarkably, the aptasensor could quantitatively monitor cocaine with an ultra-low detection limit.

Supplementary Table S1 indicates a comparison between the performance of the developed aptasensor and some available aptasensors for the cocaine detection. The designed aptasensor embedded in the capillary tube has the high sensitivity to cocaine in comparison with some other aptasensors. In general, other aptasensors can detect cocaine at the picomolar or nanomolar levels, whereas the developed aptasensor can precisely determine cocaine from picomolar to micromolar levels. Moreover, the LOD of the designed aptasensor is at the picomolar level, shown its ultra-sensitivity. Although it is obtained less LOD value based on the other study, the designed aptasensor provides the dramatically extensive linear concentration range. The other aptasensors based on electrochemical and fluorescence methods are restricted by the expensive apparatus and long term preparation.

As a result, the aptasensor embedded in the capillary tube is applicable for the on-site target detection just by using low amount of the sample. There are some drawbacks in the constructed aptasensor like the yellow points in the microscopic photos that may be due to the lack of the flat surface in the internal surface of the capillary tube. So, the deficiency should be modified for future practical application.

### 3.4 Aptasensor selectivity

**FIGURE 4:**
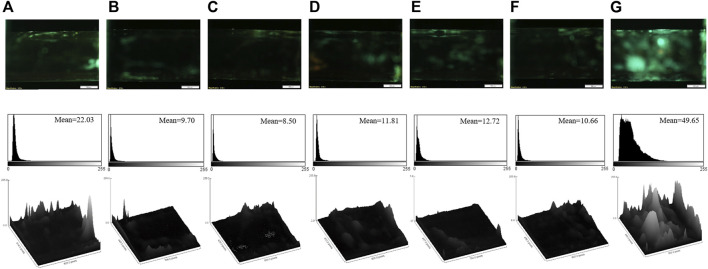
The fluorescence images, corresponding mean brightness values, and surface plots of the aptasensor in the presence of the different drugs: **(A)** diazepam; **(B)** lorazepam; **(C)** methadone; **(D)** morphine; **(E)** tramadol; **(F)** ibuprofen; **(G)** cocaine (the final concentration of each compound is 8 μM). Scale bar: 500 μm.

The selectivity of the aptasensor was confirmed by evaluating its response in the presence of some addictive drugs, such as morphine, tramadol, methadone, ibuprofen, diazepam, and lorazepam. The fluorescence images of the aptasensor, corresponding mean brightness values, and surface plots in the presence of the various drugs are gathered in Figure 4. Figure 4 clarifies that the other drugs could not enhance the fluorescence response of the aptasensor, because of non-specific affinity for the aptamer binding. Hence, the sensitive aptasensing platform possessed a superior selectivity toward cocaine.

### 3.5 Real sample monitoring

The practical efficiency of the aptasensor was assessed in the real samples (human serum and urine) by spiking cocaine in these matrices. The initial non-spiked samples were tested by the aptasensor. For more confirmation, the non-spiked human serum samples were also tested by HPLC method. The results indicated that the initial concentration of cocaine in the real samples was zero. Then, the initial cocaine-free samples were spiked with the different concentrations of cocaine for further analysis by the aptasensor. Table 1 represents the result of the recovery values of the spiked samples in the range of 90.58%–106%. The results proved that the aptasensor can be applicable for detecting cocaine in the real samples.

**TABLE 1:** Quantification of cocaine in the real samples by the fluorescent aptasensor and HPLC analysis.

Real sample	Fluorescent aptasensor				HPLC analysis			
	Added	Found	Recovery (%)	RSD (*n* = 3)	Added	Found	Recovery (%)	RSD (*n* = 3)
Non-spiked human serum	0.0	0.0	100.0	0.11	0.0	0.0	100.0	0.05
Human serum 1	0.5 nM	0.53 nM	106.00	0.24	0.5 mM	0.47 mM	95.5	0.04
Human serum 2	250 nM	262.23 nM	104.89	0.27	250 μM	222.5 μM	89	0.07
Human serum 3	50 μM	48.18 μM	96.36	0.21	50 μM	49.6 μM	99.3	0.11
Non-spiked urine	0.0	0.0	100.0	0.13	—	—	—	—
Urine 1	0.5 nM	0.46 nM	92.00	0.19	—	—	—	—
Urine 2	250 nM	243.15 nM	97.26	0.20	—	—	—	—
Urine 3	50 μM	45.29 μM	90.58	0.18	—	—	—	—

To confirm the aptasensor functionality for the cocaine detection in the real samples, some samples were analyzed by the aptasensor and compared with the HPLC results (Table 1). Cocaine solutions with the concentrations of 5 mM, 250 μM, and 50 μM were analyzed by using HPLC technique as the spiked samples. Based on Table 1, the recovered concentrations were measured as 0.47 mM, 222.5, and 49.6 μM with the recoveries of 95.5%, 89%, and 99.3%, respectively. Besides, cocaine with the concentrations of 0.5 and 250 nM could not be detected by the HPLC technique. The dilution factors of 10^6^ and 10^3^ was applied for the samples with the concentrations of 5 mM and 250 μM, respectively, to be detectable by the designed aptasensor. Since the developed aptasensor could detect cocaine with the LOD of 0.31 pM, it is a highly sensitive approach compared to the HPLC method.

To evaluate the stability of the aptasensor, the fluorescence microscopic images of the aptamer were recorded at the moment of its embedding in the capillary tube and after passing 24 h. The images were analyzed by ImageJ software, and the results are given in Supplementary Figure S10. It was also done for the aptasensor after the incubation with cocaine (600 μM, 3 μl) at the optimum incubation time and after passing 24 h. Supplementary Figure S10 indicates a 4% decrease in the fluorescence intensity of the aptasensor. Thus, the developed aptasensor is stable for the application during 24 h.

## 4 Conclusion

To sum up, a simple fluorescent aptasensor was fabricated by using capillary tube as the sensing substrate to determine the low amounts of cocaine, for the first time. The aptasensor was designed by immobilizing the dye-functionalized aptamer on the internal surface of the APTES-coated capillary tube. The fluorescence response of the aptamer was quenched by adding AuNPs through forming the aptamer@AuNPs complex. With adding cocaine, a high tendency of the aptamer toward the target released AuNPs by perturbing the complex that subsequently recovered the fluorescence response of the aptasensor. The aptasensor could dramatically monitor cocaine with a remarkable concentration range of 100 pM–600 µM and a significant LOD of 0.31 pM. Moreover, the aptasensor could successfully quantify cocaine in the real samples, e.g. human serum and urine. The developed aptasensor is cost-effective tool just by using a volume of sample about 3 μl, which makes it more significant than some other available aptasensors. The aptasensor can be a portable detection tool by using a handheld single-beam fluorescence microscope.

## Data Availability

The original contributions presented in the study are included in the article/Supplementary Materials, further inquiries can be directed to the corresponding authors.
