# Supplementary material for: An ultrasensitive detection platform for cocaine: Aptasensing strategy in capillary tube
Source: Front Chem. 2022 Oct 19;10:996358. doi: 10.3389/fchem.2022.996358 (PMC9626653; doi:10.3389/fchem.2022.996358)
Supplement: Supplementary file 1 [file DataSheet1.docx]

Supporting information

**An Ultrasensitive Detection Platform for Cocaine: Aptasensing Strategy in Capillary Tube**

Javad Zamanian^a,b,e^ , Zahra Khoshbin^a,b,e^ , Hossein Hosseinzadeh^a,c^, Noor Mohammd Danesh^b,d^, Ali khakshour Abdolabadi^b^, Khalil Abnous^a,b,*^, Seyed Mohammad Taghdisi^e,f,*^

^a^ Pharmaceutical Research Center, Pharmaceutical Technology Institute, Mashhad University of Medical Sciences, Mashhad, Iran

^b^ Department of Medicinal Chemistry, School of Pharmacy, Mashhad University of Medical Sciences, Mashhad, Iran

^c^ Department of Pharmacodynamics and Toxicology, School of Pharmacy, Mashhad University of Medical Sciences, Mashhad, Islamic Republic of Iran

^d^ Institute of Science and New Technologies, Tehran, Iran

^e^ Targeted Drug Delivery Research Center, Pharmaceutical Technology Institute, Mashhad University of Medical Sciences, Mashhad, Iran

^f^ Department of Pharmaceutical Biotechnology, School of Pharmacy, Mashhad University of Medical Sciences, Mashhad, Iran

* Corresponding authors:

**Prof. Khalil Abnous**, Professor of Department of Medicinal Chemistry, Pharmaceutical Research Center, School of Pharmacy, Mashhad University of Medical Sciences, Mashhad, Iran. Tel.: +98 5131801112, Fax: +98 5138823251, E-mail: [abnouskh@mums.ac.ir](mailto:abnouskh@mums.ac.ir)

**Dr. Seyed Mohammad Taghdisi**, Associate Professor of Department of Pharmaceutical Biotechnology, Targeted Drug Delivery Research Center, School of Pharmacy, Mashhad University of Medical Sciences, Mashhad, Iran. Tel.: +98 5131801203, Fax: +98 5138823251, E-mail: taghdisihm@mums.ac.ir


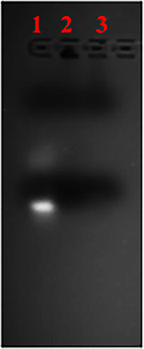


**Figure S1.** Confirmation of the formation of the aptasensor containing aptamer and AuNPs by using agarose gel electrophoresis. Lane 1: Specific aptamer, Lane 2: AuNPs, Lane 3: Aptamer@AuNPs complex.


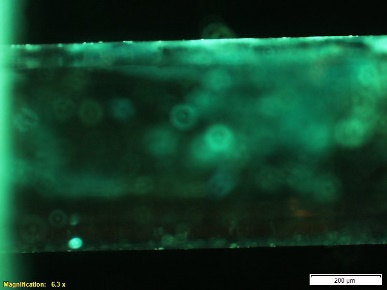

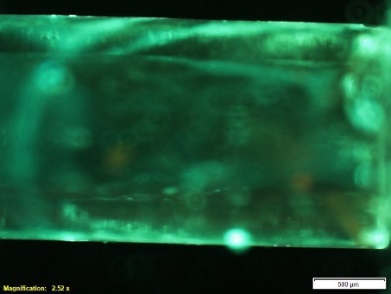

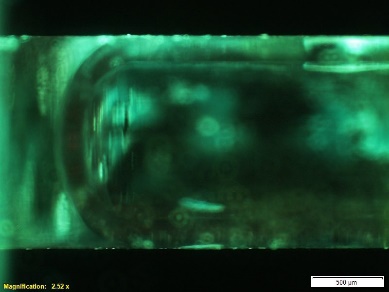

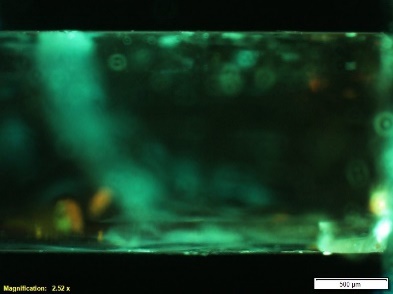

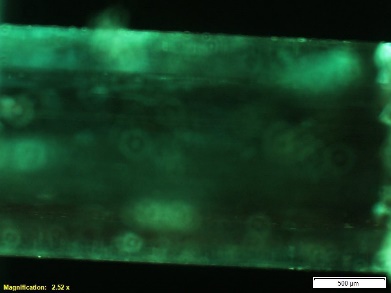

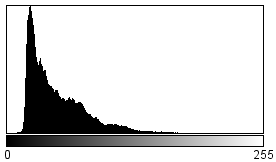

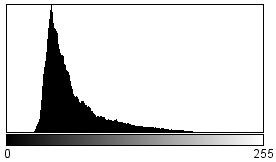

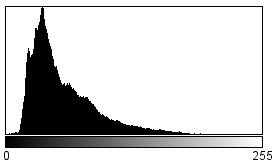

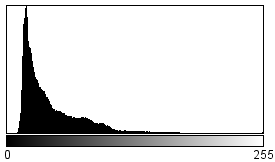

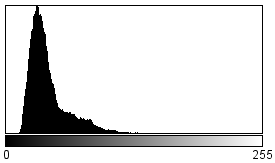

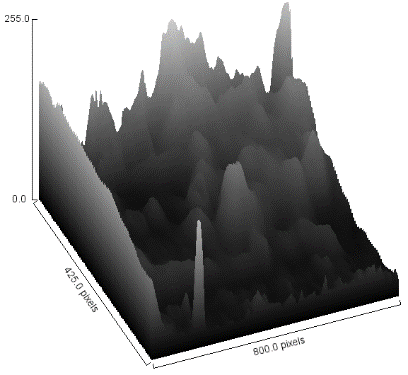

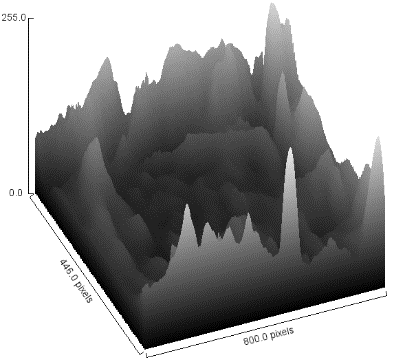

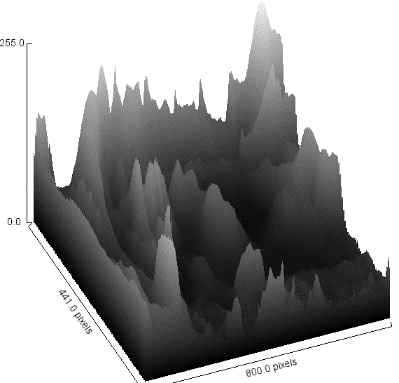

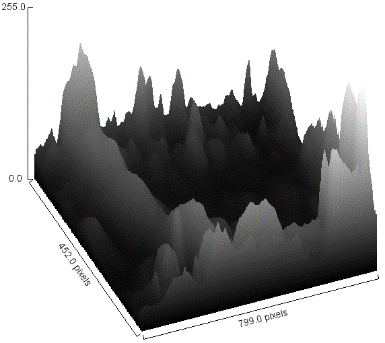

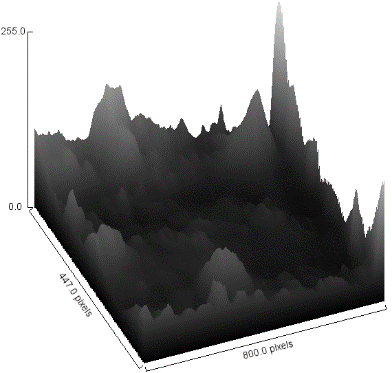

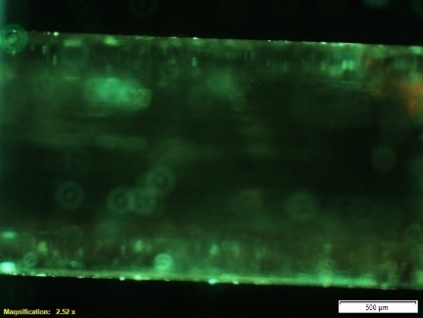

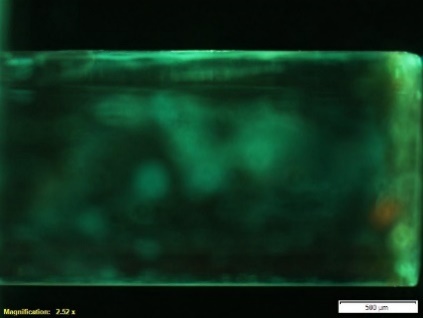

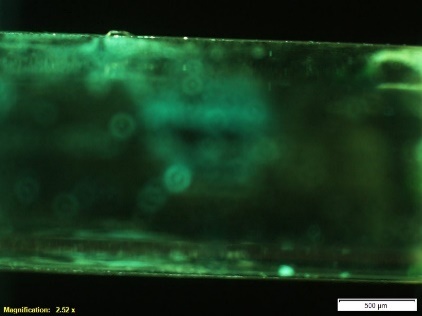

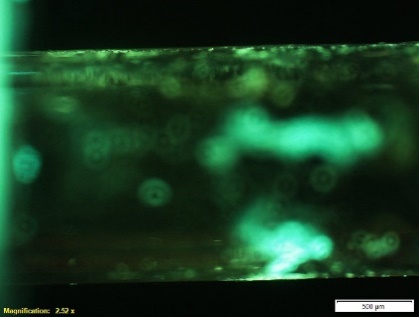

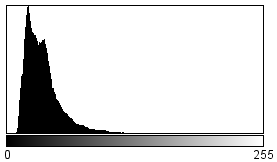

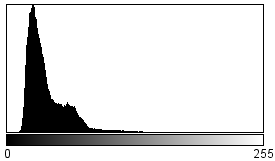

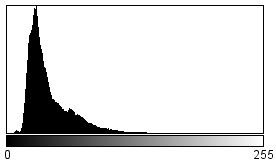

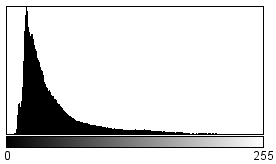

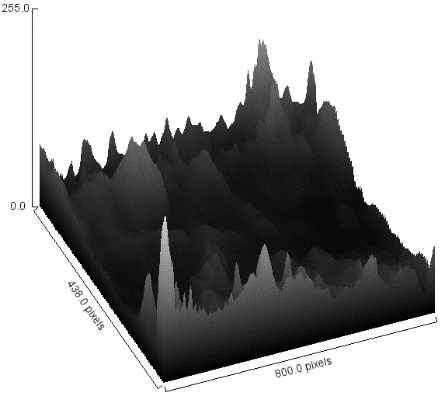

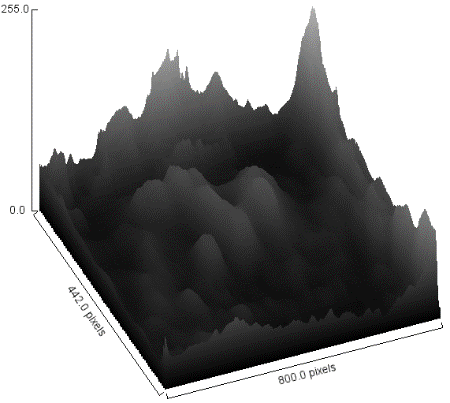

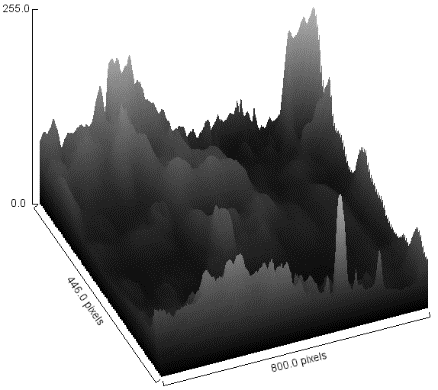

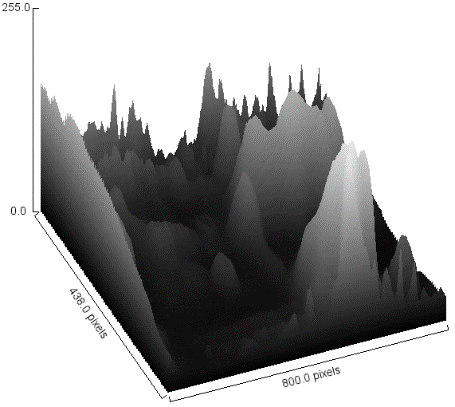


**Figure S2. (A).** The fluorescence images, corresponding mean brightness values, and surface plots of the capillary tubes modified by the different APTES and glutaraldehyde concentrations (% (v/v)): (A) 1.8 & 0.9; (B) 1.8 & 1.8; (C) 1.8 & 2.7; (D) 3.6 & 0.9; (E) 3.6 & 1.8; (F) 3.6 & 2.7; (G) 5.4 & 0.9; (H) 5.4 & 1.8; (I) 5.4 & 2.7. Scale bar: 500 μm.

**Figure S2. (B).** Plot of the mean brightness value of the aptasensor versus the different concentrations of APTES and glutaraldehyde. The error bars represent the average standard errors for three measurements.


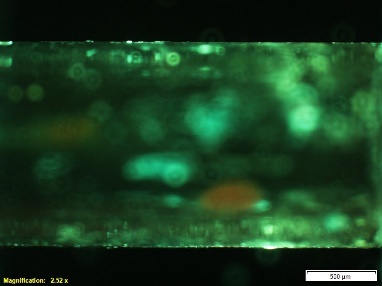

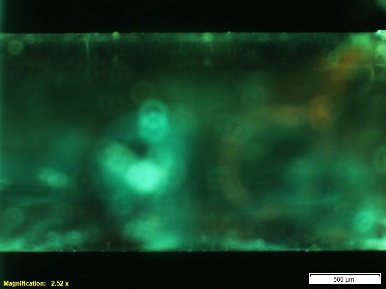

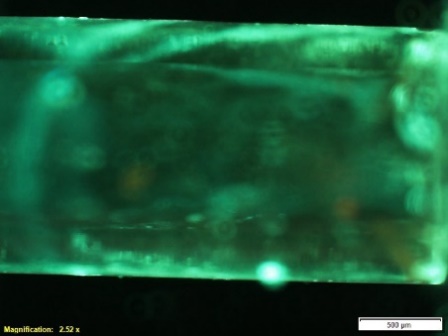

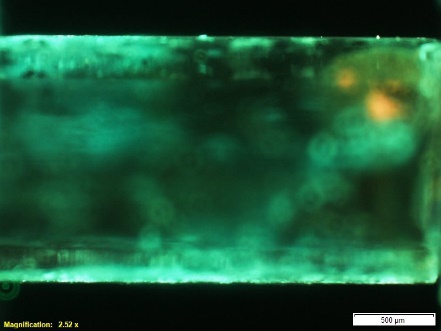

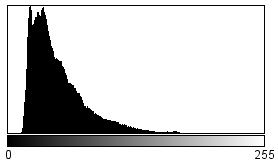

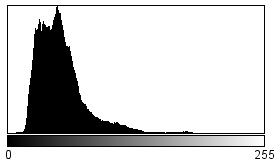

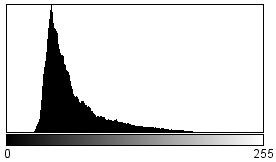

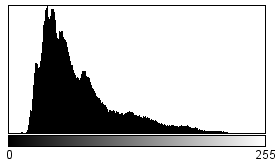

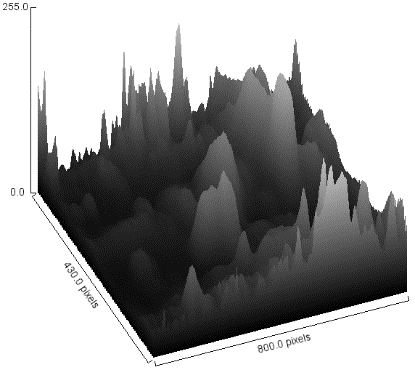

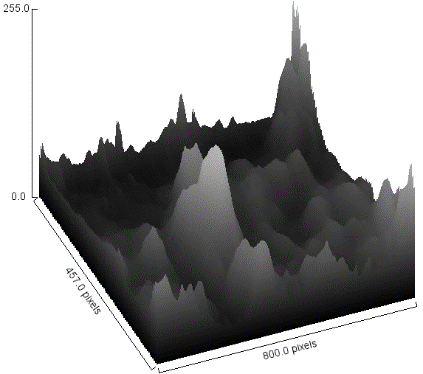

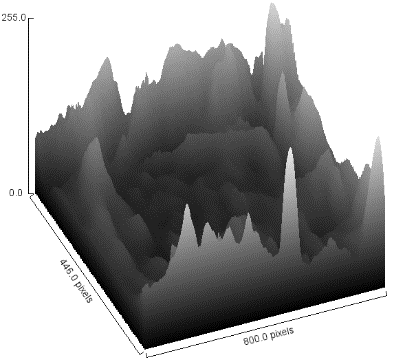

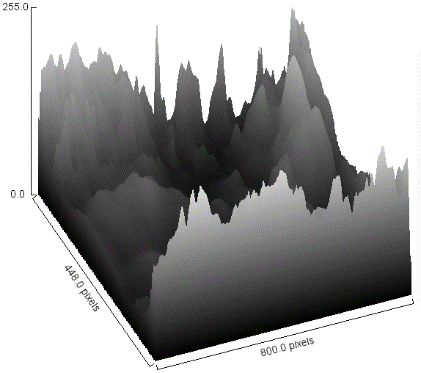


**Figure S3. (A).** The fluorescence images, corresponding mean brightness values, and surface plots of the capillary tubes modified by the different aptamer concentrations: (A) 100 nM; (B) 200 nM; (C) 500 nM; (D) 700 nM. Scale bar: 500 μm.

**Figure S3. (B).** Plot of the mean brightness value of the aptasensor versus the different concentrations of the aptamer strand. The error bars represent the average standard errors for three measurements.


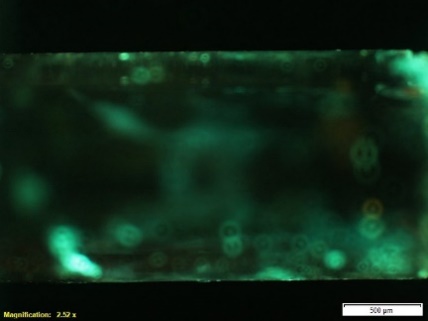

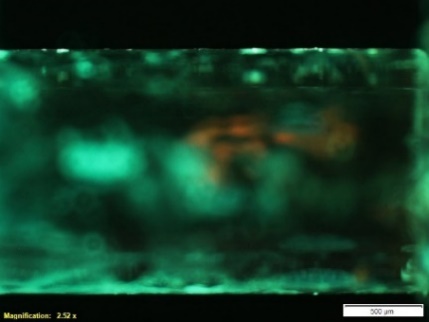

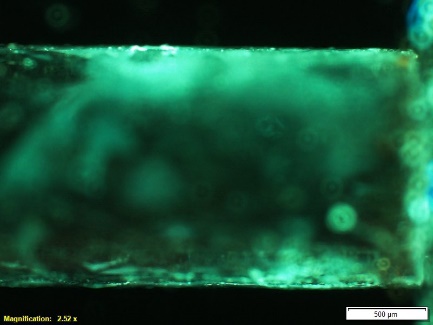

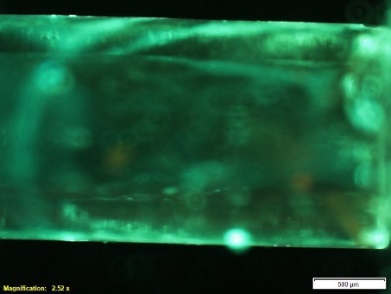

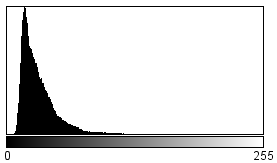

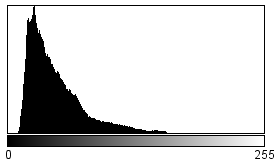

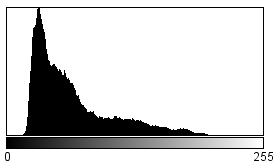

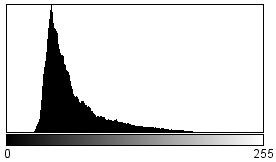

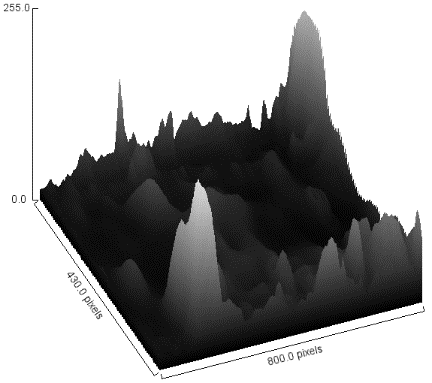

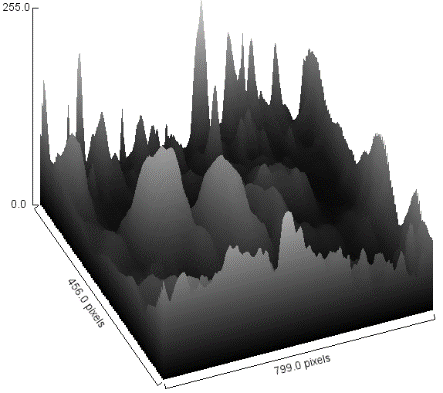

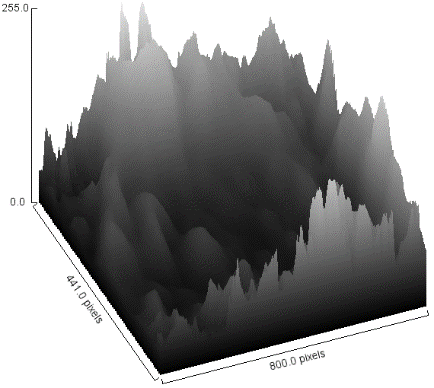

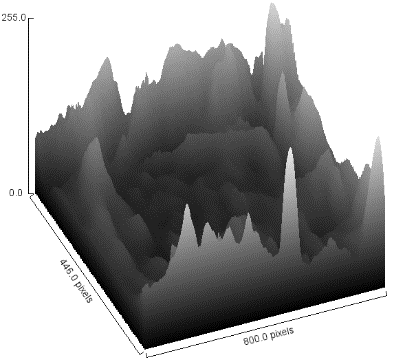


**Figure S4. (A).** The fluorescence images, corresponding mean brightness values, and surface plots of the capillary tubes containing 1.8% (v/v) of APTES, 1.8% (v/v) of glutaraldehyde, and 500 nM of the aptamer at the different incubation times: (A) 0.5 h; (B) 1 h; (C) 1.5 h; (D) 2 h. Scale bar: 500 μm.

**Figure S4. (B).** Plot of the mean brightness value of the aptasensor versus the different incubation times of the aptamer strand. The error bars represent the average standard errors for three measurements.


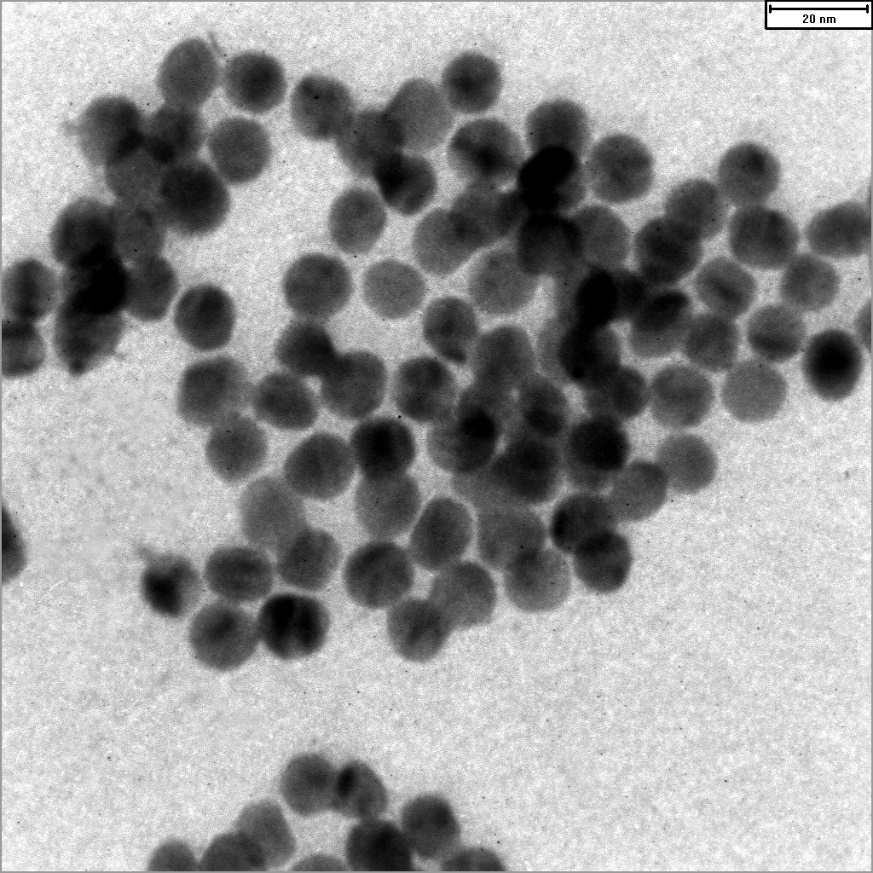


**Figure S5.** TEM image of the synthesized AuNPs.


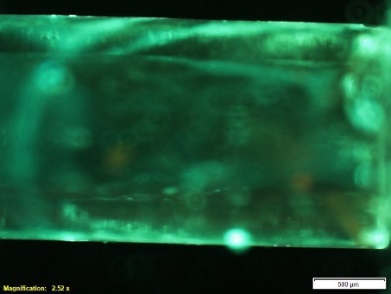

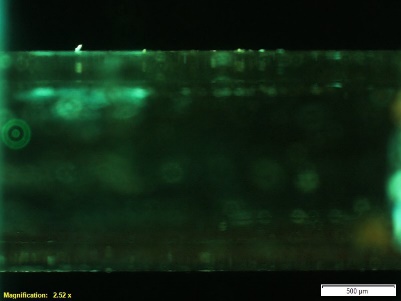

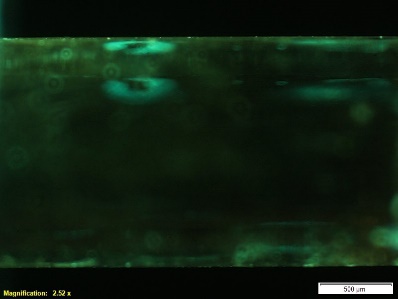

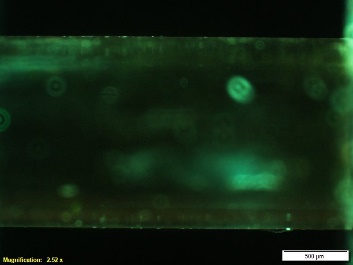

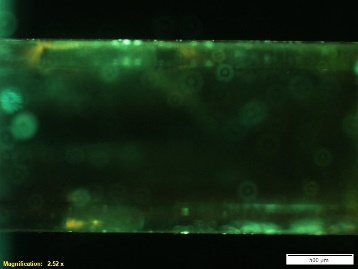

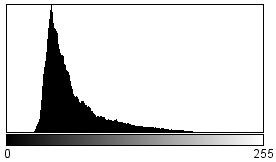

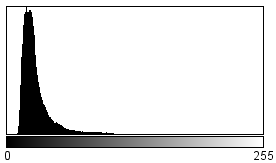

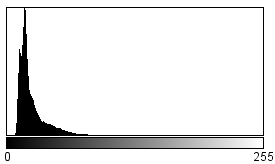

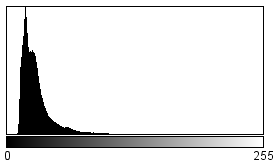

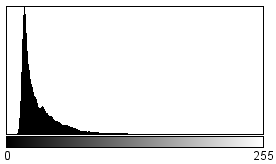

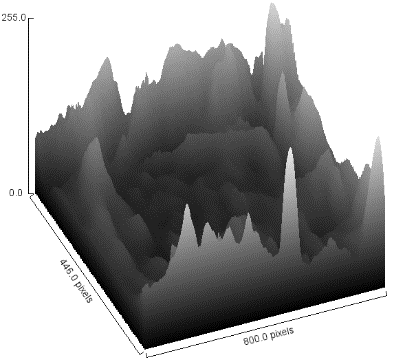

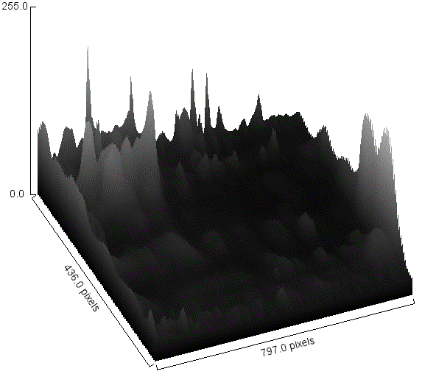

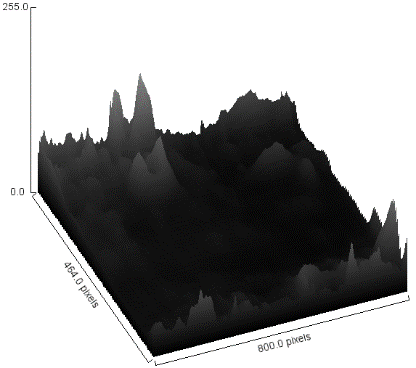

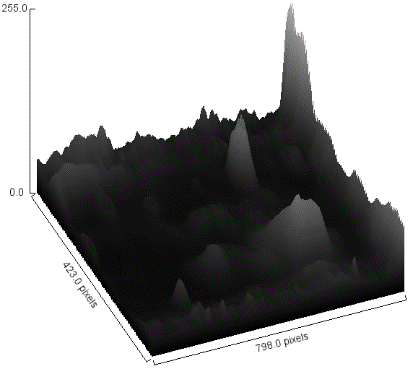

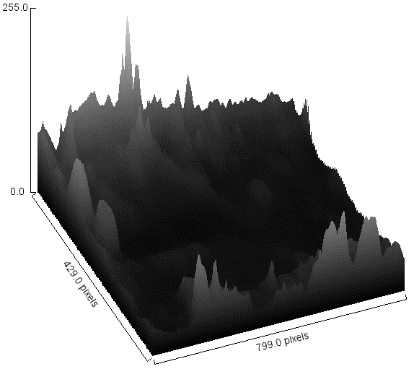

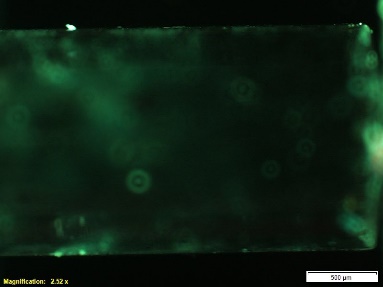

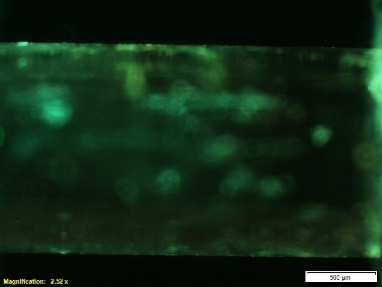

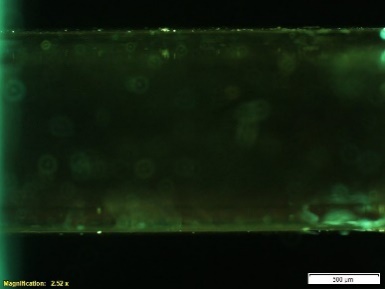

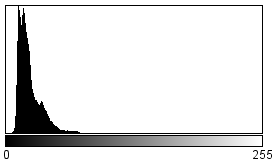

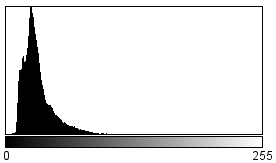

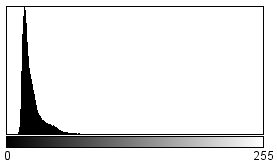

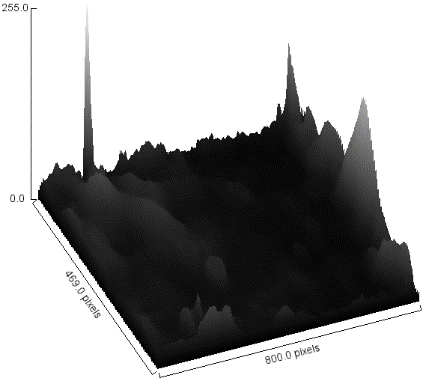

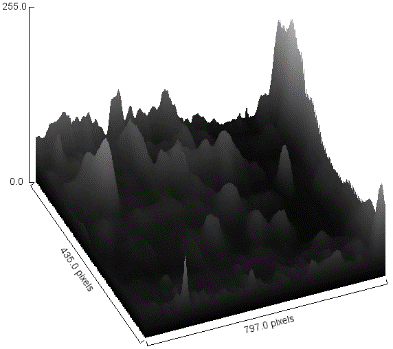

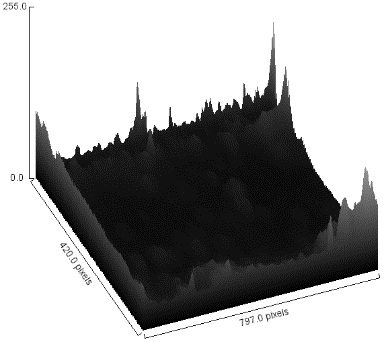


**Figure S6. (A).** The fluorescence images, corresponding mean brightness values, and surface plots of the aptasensor in the absence (A) and presence of the different nanoquenchers: (B) SNPs; (C) CNT; (D) GO; (E) activated carbon; (F) AuNPs; (G) CuNPs; (H) AgNPs. Scale bar: 500 μm.

**Figure S6. (B).** Plot of the mean brightness value of the aptasensor versus the different quenchers. The error bars represent the average standard errors for three measurements.


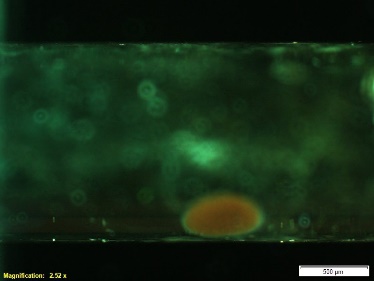

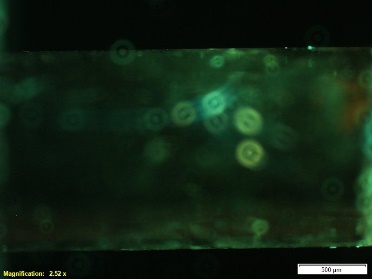

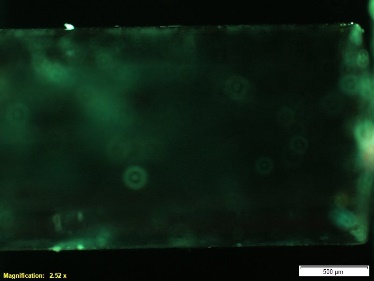

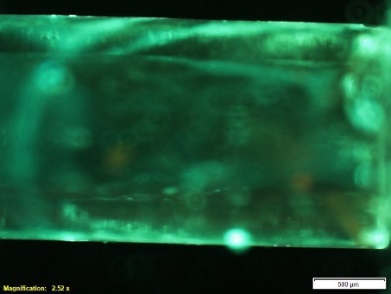

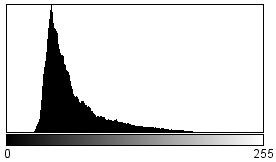

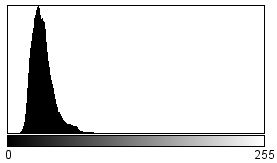

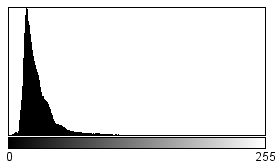

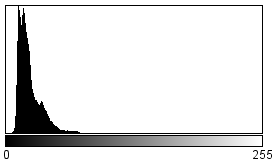

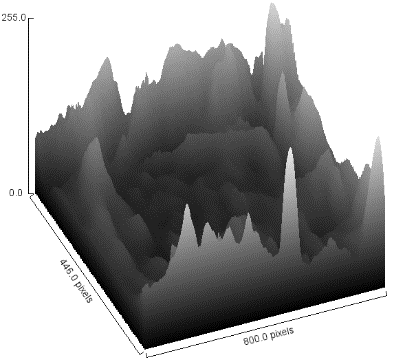

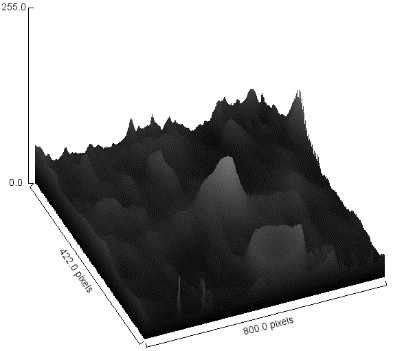

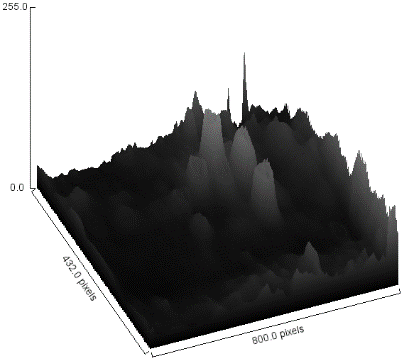

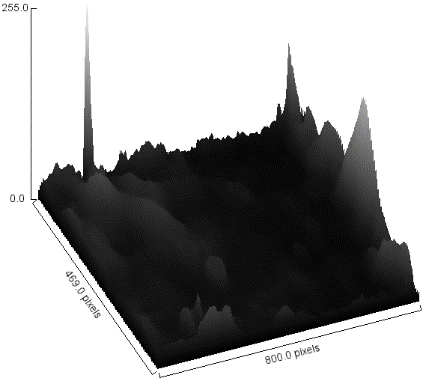


**Figure S7. (A).** The fluorescence images, corresponding mean brightness values, and surface plots of the aptamer-modified capillary tubes in the absence (A) and presence of the different concentrations of AuNPs: (B) 0.2 nM; (C) 0.4 nM; (D) 0.6 nM; (E) 1 nM. Scale bar: 500 μm.

**Figure S7. (B).** Plot of the mean brightness value of the aptasensor versus the different concentrations of AuNPs. The error bars represent the average standard errors for three measurements.


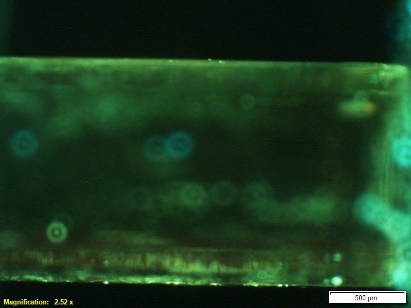

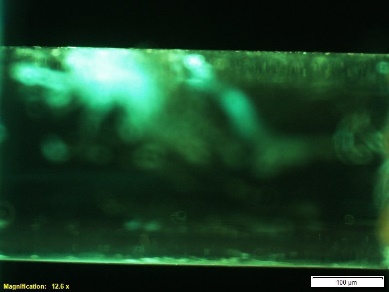

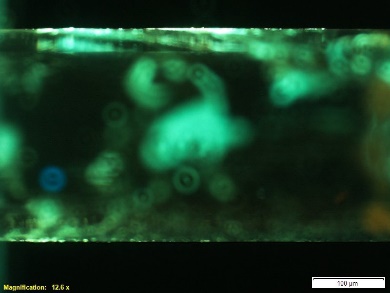

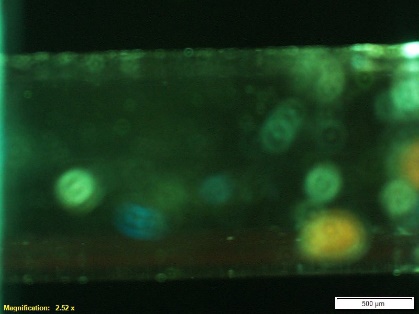

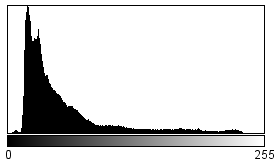

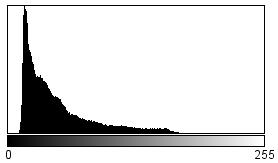

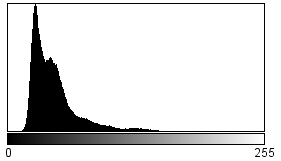

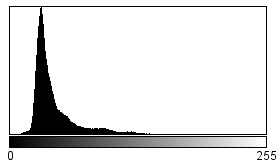

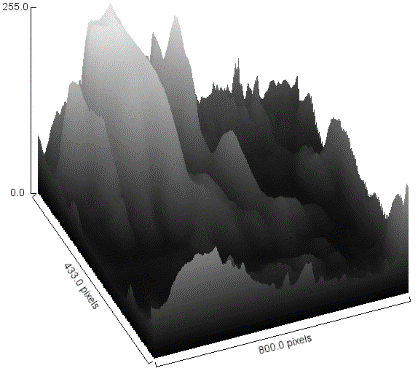

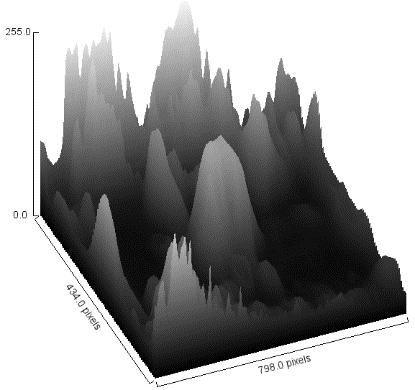

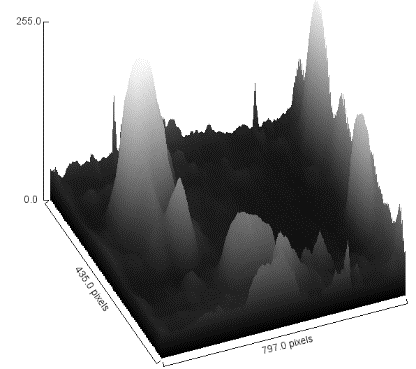


**Figure S8. (A).** The fluorescence images, corresponding mean brightness values, and surface plots of the capillary tubes containing the aptamer@AuNPs complex at the different incubation times of AuNPs: (A) 15 min; (B) 30 min; (C) 45 min; (D) 60 min; (E) 75 min: (F) 90 min; (G) 105 min; (H) 120 min. Scale bar: 500 μm.

**Figure S8. (B).** Plot of the mean brightness value of the aptasensor versus the different incubation times of AuNPs. The error bars represent the average standard errors for three measurements.

**Figure S9. (A).** The fluorescence images, corresponding mean brightness values, and surface plots of the aptasensor in the presence of cocaine (600 µM) at the different incubation times: (A) 0.5 h; (B) 1 h; (C) 1.5 h; (D) 2 h. Scale bar: 500 μm.

**Figure S9. (B).** Plot of the mean brightness value of the aptasensor versus the different incubation times of cocaine. The error bars represent the average standard errors for three measurements.

**Figure S10.** Plot of the mean brightness value of the aptasensor at the moment of its embedding in the capillary tube and after passing 24 h (Green); and the aptasensor after the incubation with cocaine (600 µM, 3 µL) at the optimum incubation time and after passing 24 h (Blue). The error bars represent the average standard errors for three measurements.

| **Table S1.** Comparison between the available aptasensing methods and the designed aptasensor for the Cocaine detection. | | | | | | |  |
| --- | --- | --- | --- | --- | --- | --- | --- |
| **No.** | **Method** | **Detection Strategy** | **Detection Limit** | **Linear Range** | **Real Sample** | **Reference** | |
| 1 | Electrochemical impedance spectroscopy | Coating aptamer on the surface of imprinted polymer nanoparticles attaching onto the gold electrode | 0.70 nM | 0.30-147 nM | Not reported | (1) | |
| 2 | Electrochemical | Functionalizing aptamer on nanoporous gold electrode | 21 nM | 0.05-1 and 1-35 mM | Not reported | (2) | |
| 3 | Electrochemical impedance spectroscopy | Immobilizing aptamer on the screen-printed electrode modified by nanocomposite and silver nanoparticles (AgNPs) | 333 aM | 1 FM-100 nM | Human serum | (3) | |
| 4 | Colorimetry | Forming three-way junction pockets structure on the surface gold nanoparticles | 440 pM | 2-100 nM | Human serum | (4) | |
| 5 | Colorimetry | Aggregation of gold nanoparticles in the presence of NaCl | 0.97 nM | 118 nM- 55 mM | Saliva | (5) | |
| 6 | Fluorescence | Immobilizing aptamer on the silica nanoparticles coated with streptavidin | 84 pM | 500 pM-80 nM | Human serum | (6) | |
| 7 | Fluorescence | Combination of the FAM-labeled specific aptamer and AuNPs in a capillary tube as the sensing substrate | 0.31 pM | 100 pM-600 µM | Human serum and urine | This Study | |

**References:**

1. D'Aurelio R, Chianella I, Goode JA, Tothill IE. Molecularly Imprinted Nanoparticles Based Sensor for Cocaine Detection. Biosensors (Basel). 2020;10(3).

2. Tavakkoli N, Soltani N, Mohammadi F. A nanoporous gold-based electrochemical aptasensor for sensitive detection of cocaine. RSC Advances. 2019;9(25):14296-301.

3. Roushani M, Shahdost-Fard F. Impedimetric detection of cocaine by using an aptamer attached to a screen printed electrode modified with a dendrimer/silver nanoparticle nanocomposite. Mikrochim Acta. 2018;185(4):214.

4. Abnous K, Danesh NM, Ramezani M, Taghdisi SM, Emrani AS. A novel colorimetric aptasensor for ultrasensitive detection of cocaine based on the formation of three-way junction pockets on the surfaces of gold nanoparticles. Anal Chim Acta. 2018;1020:110-5.

5. Sanli S, Moulahoum H, Ghorbanizamani F, Celik EG, Timur S. Ultrasensitive covalently-linked Aptasensor for cocaine detection based on electrolytes-induced repulsion/attraction of colloids. Biomed Microdevices. 2020;22(3):51.

6. Abnous K, Danesh NM, Ramezani M, Taghdisi SM, Emrani AS. A novel amplified double-quenching aptasensor for cocaine detection based on split aptamer and silica nanoparticles. Analytical Methods. 2018;10(26):3232-6.
